# Supplementary material for: Flotillins promote T cell receptor sorting through a fast Rab5–Rab11 endocytic recycling axis
Source: Nat Commun. 2019 Sep 26;10:4392. doi: 10.1038/s41467-019-12352-w (PMC6763463; doi:10.1038/s41467-019-12352-w)
Supplement: Supplementary file 1 — Supplementary information [file 41467_2019_12352_MOESM1_ESM.pdf]

Flotillins promote T cell receptor sorting through a fast Rab5-Rab11 endocytic recycling axis

G.M.I. Redpath et al.

SUPPLEMENTARY INFORMATION

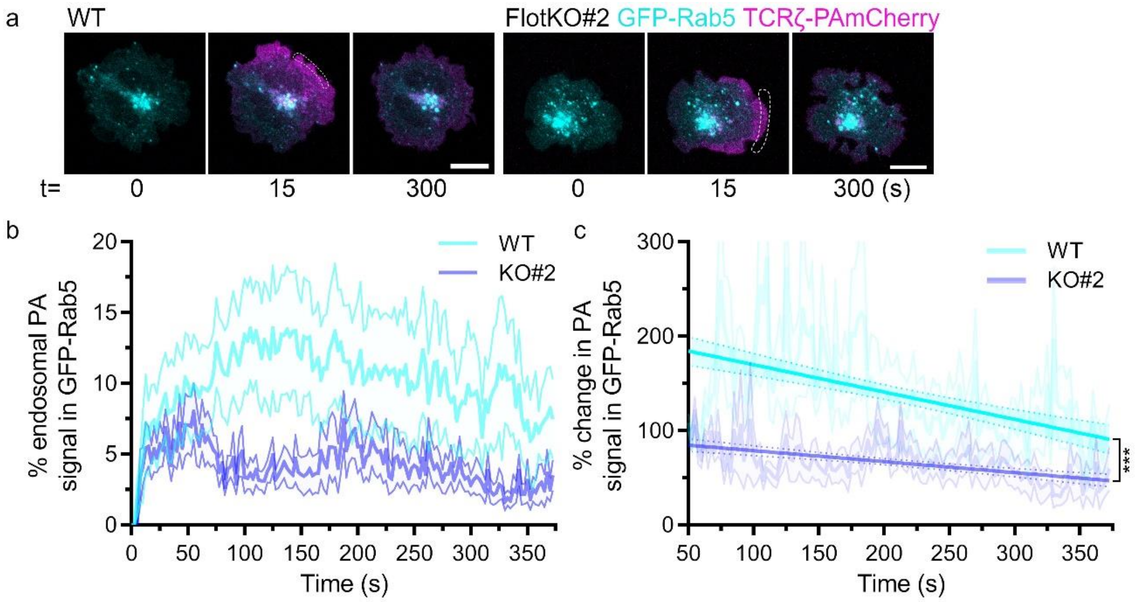

Figure S1: Flotillins mediate sorting of internalised TCRζ into Rab5 in a second flotillin knockout cell line. (a) Representative images of TCRζ-PAmCherry photoactivated at the dashed regions sorting into EGFP-Rab5 endosomes in WT (left panel) and FlotKO (right panel) Jurkat T cells. Cells were photoactivated for 5 frames with 2.5 second intervals, then imaged every 2.5 seconds for 150 frames. **(b)** % endosomal TCRζ-PAmCherry intensity present in Rab4 endosomes in WT or FlotKO Jurkat T cells over time. Dashed line represents the timepoint from which the TCRζ-PAmCherry intensity significantly diverges in Rab4 endosomes between WT and FlotKO. **(c)** Percentage reduction in endosomal TCRζ-PAmCherry intensity in Rab5 endosomes from the 50-55 second timepoint. Fitted linear regression line is in bold. n.s. indicates differences between slopes are not significantly different. *** indicates slopes are highly significantly different. Data points= means of 3 independent experiments (WT: 3; 5; 5 cells, KO#2: 3; 5; 4 cells per experiment), error bars= mean± SEM. All images acquired on a Zeiss 880 confocal microscope. Scale bar = 5µm.

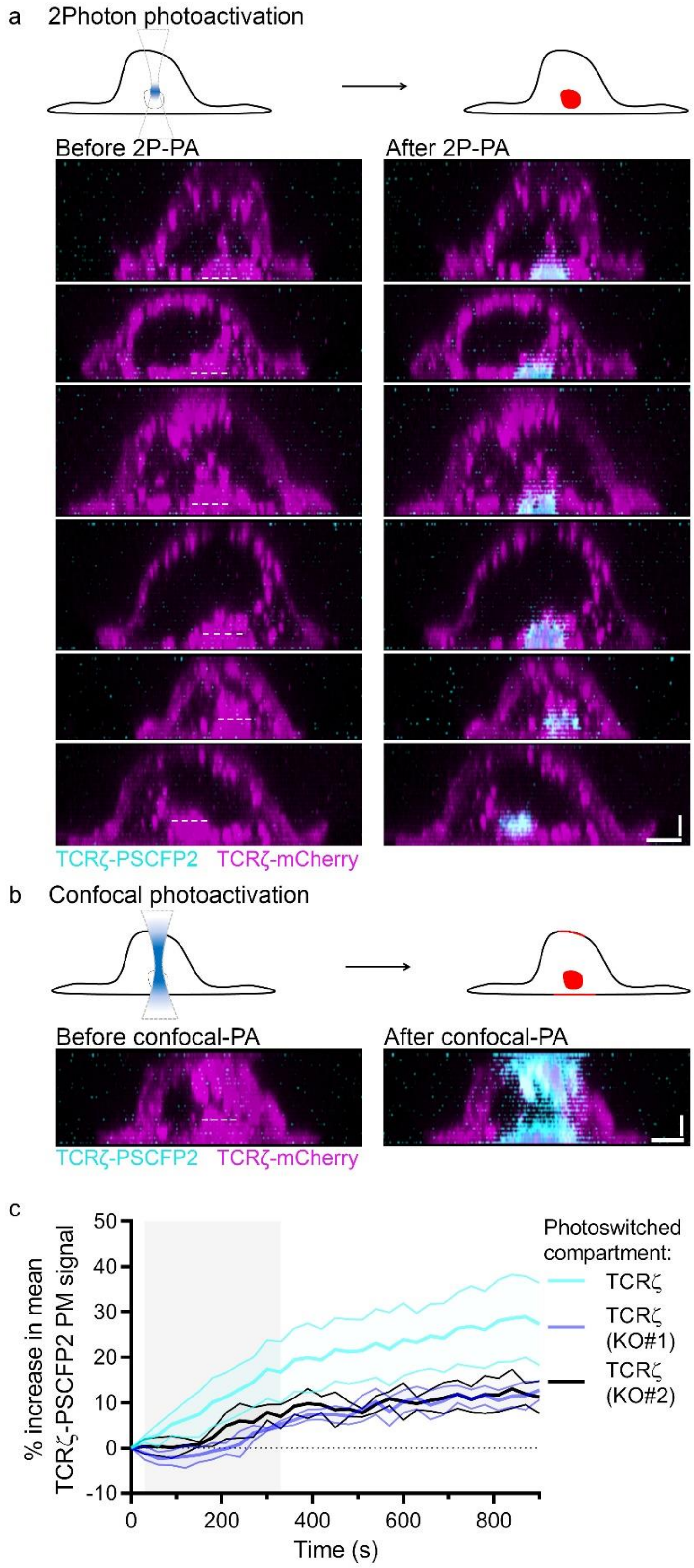

Figure S2: Two-photon photoactivation in fixed cells. (a) Jurkat T cells transfected with TCRζ-mCherry (magenta) and TCRζ-PSCFP2 (cyan) were activated on glass coverslips and fixed 20 minutes post activation. Z-stacks of cells were imaged before (left) and after (right) photoactivation with 800 nm light from a two-photon laser at the indicated z-depth. Dashed white line indicates photoactivated region. **(b)** Confocal photoactivation at 2.5 μm z-depth of cells prepared as in **(a)**. **(c)** % increase in photoactivated TCRζ-PSCFP2 signal over time at the plasma membrane when activated from cell centre compartments labelled by TCRζ-mCherry in WT or two FlotKO Jurkat T cells lines. Greyed region= time of photoactivation. Horizontal scale bars = 5 μm, vertical = 2 μm. Error bars= mean± SEM from 1 experiment of 3 (WT); 2 (FlotKO#2) and 2 (FlotKO#1) cells.

a WT Jurkat T cells, TCRζ-GFP + APEX2-GBP

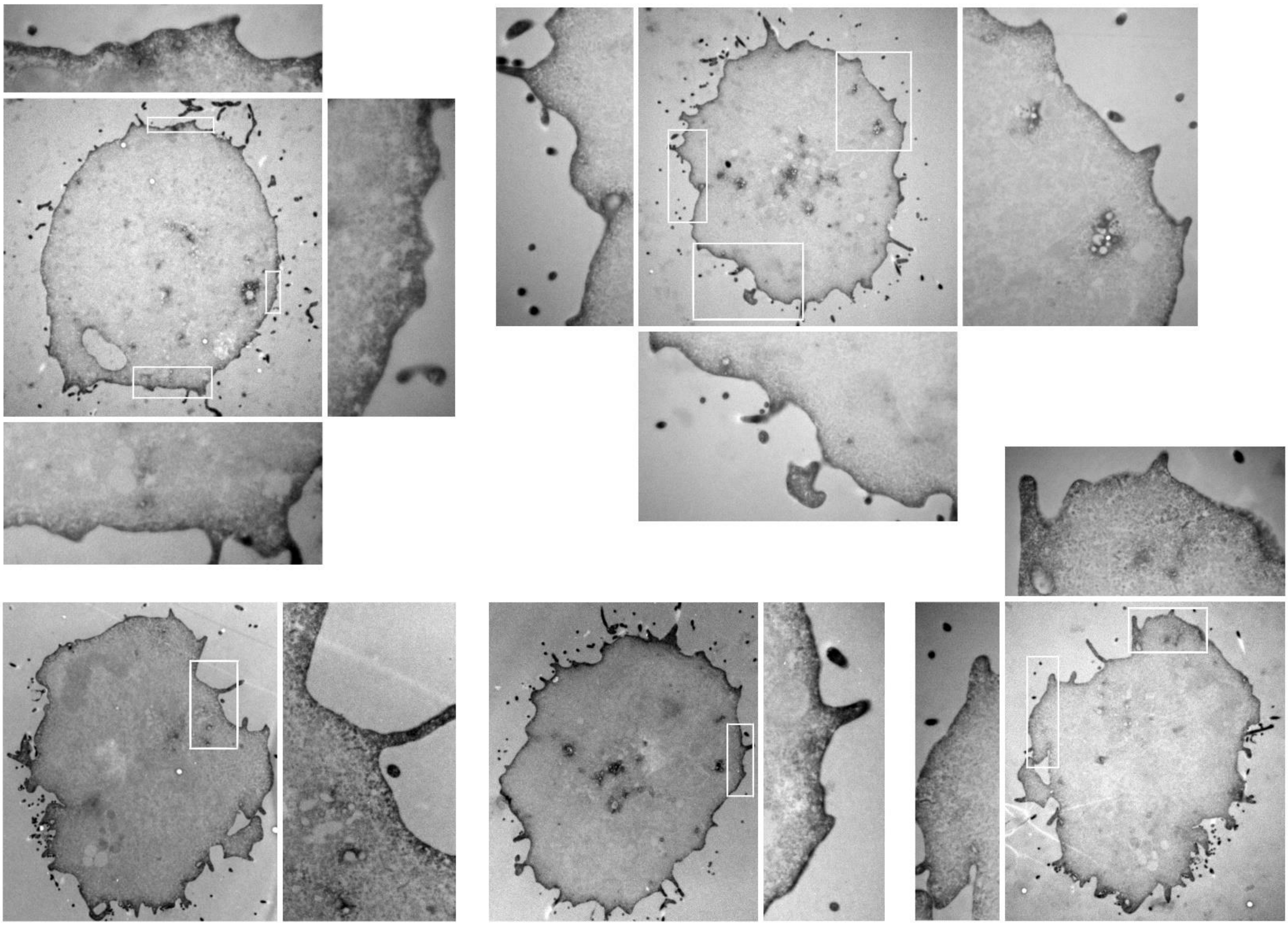

b Flotillin1/2 KO Jurkat T cells, TCRζ-GFP + APEX2-GBP

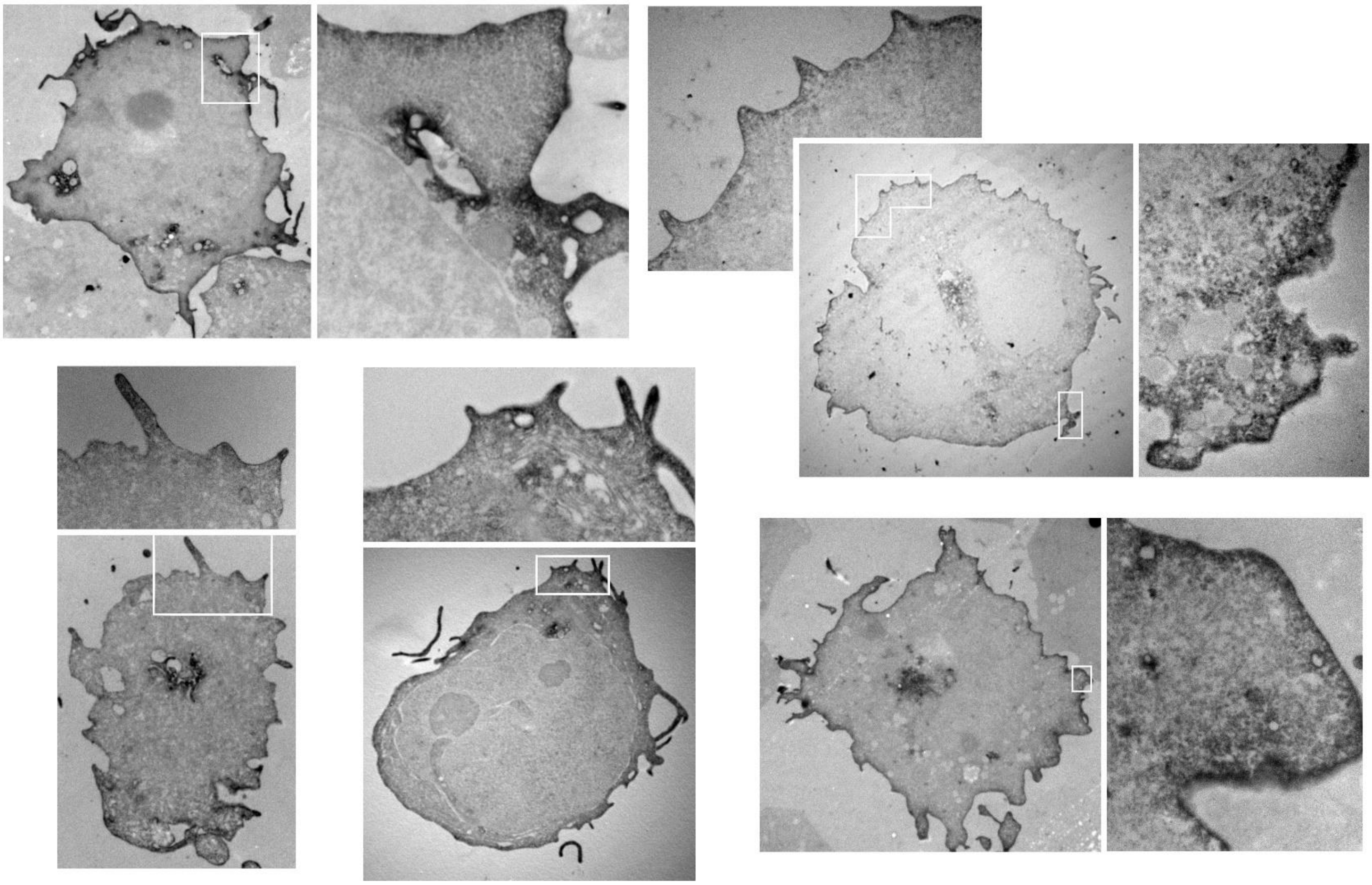

Figure S3: Flotillins do not regulate plasma membrane morphology of Jurkat T cells. (a) Transmission electron microscopy images of WT Jurkat T cells with DAB-reacted APEX2-GBP co-expressed with TCRζ-GFP. Images are obtained from 3 (WT) or 4 (FlotKO#1) biologically independent experiments (WT: 4; 3; 1 cells per experiment) and 4 (FlotKO#1: 7; 1; 2; 2 cells per experiment) **(b)** Transmission electron microscopy images of flotillin 1 and 2 knockout Jurkat T cells with DAB-reacted APEX2-GBP co-expressed with TCRζ-GFP. White boxes denote insets.

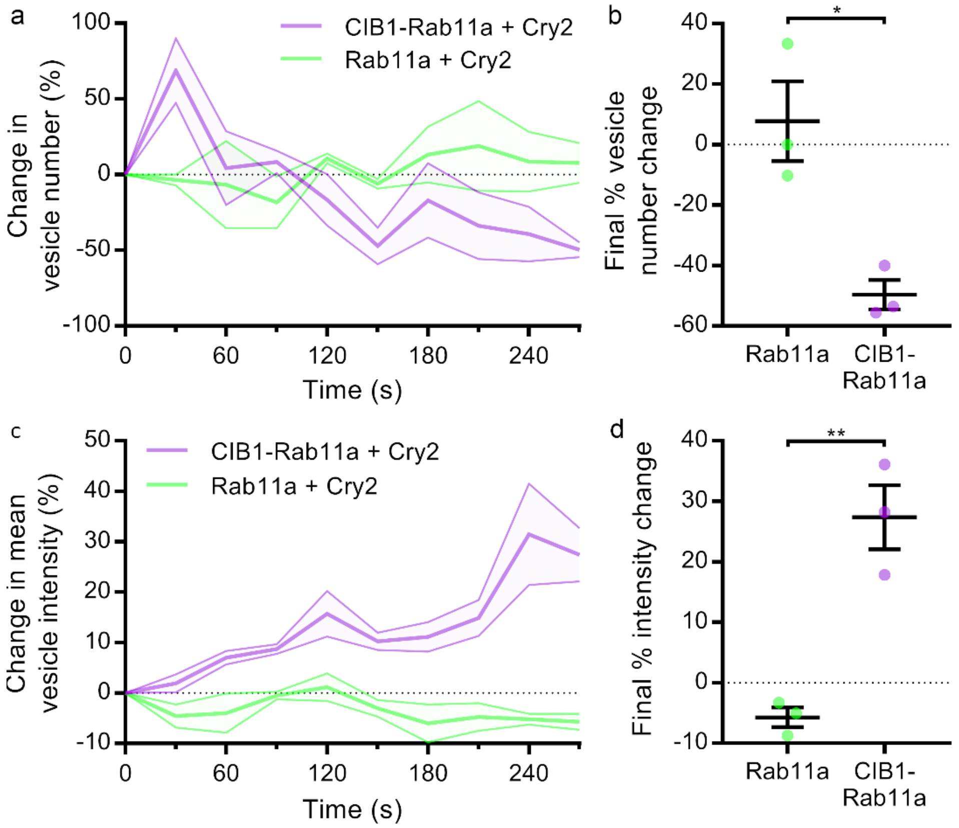

Figure S4: Acute exposure to blue light induces Cry2clust-mediated CIB1-Rab11 clustering. Jurkat T cells transfected with mCerulean-Cry2clust, TCRζ-PSCFP2 and either mCherry-Rab11a or CIB1-FugeneRed-Rab11a were exposed to 488 nm light by confocal imaging for three frames through the z plane every 30 seconds for 10 iterations. **(a)** Quantification of the percentage change in CIB1-Rab11a and Rab11a vesicle number over time with 488 nm light exposure. **(b)** Percentage change in CIB1-Rab11a and Rab11a vesicle number at the final timepoint of 488 nm light exposure. **(c)** Quantification of the percentage change in CIB1-Rab11a and Rab11a vesicle mean intensity over time with 488 nm light exposure. **(d)** Percentage change in CIB1-Rab11a and Rab11a vesicle mean intensity at the final timepoint of 488 nm light exposure. Data points indicate means of independent experiments, error bars indicate mean± SEM. *= $p<0.05$, **= $p<0.01$ from Student's T-test of 3 independent experiments of 2; 1; 1 (mCherry-Rab11a) and 2; 1; 2 (CIB1-FugeneRed-Rab11a) cells.
