# Supplementary material for: Flotillins promote T cell receptor sorting through a fast Rab5–Rab11 endocytic recycling axis
Source: Nat Commun. 2019 Sep 26;10:4392. doi: 10.1038/s41467-019-12352-w (PMC6763463; doi:10.1038/s41467-019-12352-w)
Supplement: Supplementary file 3 — Description of Additional Supplementary Files [file 41467_2019_12352_MOESM3_ESM.docx]

**Description of Additional Supplementary Files**

**File Name: Supplementary Movie 1**

**Description:** Representative movie of TCRζ-PAmCherry sorting into Rab4, Rab5 and Rab11a compartments vesicle fusion. WT or FlotKO Jurkat T cells expressing TCRζ-PAmCherry and GFP-Rab4 or GFP-Rab5 were imaged every 2.5 seconds for 300 frames, or WT or FlotKO Jurkat T cells expressing TCRζ-PAmCherry and GFP-Rab11a were imaged every minute for 10 frames after photoactivation. The mask (white outlines) was created from the GFP-Rab signal.

**File Name: Supplementary Movie 2**

**Description:** Representative movies of photoactivated TCRζ-PSCFP2 recycling to the plasma membrane. WT Jurkat T cells expressing TCRζ-PSCFP2 (cyan) and TCRζ-mCherry, flotillin1/2-mCherry or mCheryy-Rab11a (magenta) were imaged at 30 s intervals, with two-photon photoactivation undertaken 2.5 μm within the cell in the indicated region (red circle) for the first 10 frames. Image plane is at z= 0 μm. Video plays at 10 frames per second.

**File Name: Supplementary Movie 3**

**Description:** Representative movies of TCRζ-GFP and flotillin1/2-mCherry vesicle fusion. WT Jurkat T cells co-transfected with TCRζ-GFP (left) or TCRζ-GFP (cyan) and flotillin1/2-mCherry (magenta) were imaged at 33ms (left) or 200ms intervals (right) in TIRF microscopy. Red arrows indicate TCRζ-only fusion events, yellow arrow indicates TCRζ flotillin1/2 co-fusion event. Video plays at 30 frames per second.

**File Name: Supplementary Movie 4**

**Description:** Representative movies of GFP-Rab11a or TCRζ-GFP and mCherry-Rab11a vesicle fusion. FlotKO Jurkat T cells co-transfected with GFP-Rab11a (left) or TCRζ-GFP (cyan) and mCherry-Rab11a (magenta) were imaged at 33ms (left) or 200ms intervals (right) in TIRF microscopy. Red arrows indicate TCRζ:Rab11a co-fusion events, yellow arrows indicate TCRζ only fusion events. Video plays at 30 frames per second.

**File Name: Supplementary Movie 5**

**Description:** Representative movies of flotillin2-PAmCherry sorting into Rab5 and Rab11a compartments vesicle fusion. WT Jurkat T cells expressing Flotillin2- PAmCherry, untagged flotillin-1 and GFP-Rab4, GFP-Rab5 or GFP-Rab11a were imaged every 2.5 seconds for 150 frames. Video plays at 10 frames per second.

**File Name: Supplementary Movie 6**

**Description:** Representative movie of optogenetic aggregation of Rab11a and Rab5. WT Jurkat T cells expressing TCRζ-PSCFP2 (not imaged), Cry2clust (not imaged) and either mCherry-Rab11a or CIB1-FuGeneRed-Rab11a (magenta, left panel) or TCRζPSCFP2 (not imaged), Cry2clust (not imaged) and either mCherry-Rab5 or CIB1- FuGeneRed-Rab11a (magenta, right panel) were imaged at 30 s intervals, for optogenetic aggregation for 11 frames. Image plane is at z= 0 μm. Video plays at 5 frames per second.

**File Name: Supplementary Movie 7**

**Description:** Representative movie of photoactivated TCRζ-PSCFP2 recycling to the plasma membrane following optogenetic aggregation of Rab11a and Rab5. WT Jurkat T cells expressing TCRζ-PSCFP2 (cyan), Cry2clust (not imaged) and either mCherryRab11a or CIB1-FuGeneRed-Rab11a (magenta, left panel) or TCRζ-PSCFP2 (cyan), Cry2clust (not imaged) and either mCherry-Rab5 or CIB1-FuGeneRed-Rab11a (magenta, right panel) were imaged at 30 s intervals, with two-photon photoactivation undertaken 2.5 μm within the cell in the indicated region (red circle) for = 10 frames after optogenetic aggregation for 10 frames. Image plane is at z= 1 μm. Video plays at 5 frames per second.
